# Transcranial direct current stimulation regulates phenotypic transformation of microglia to relieve neuropathic pain induced by spinal cord injury

**DOI:** 10.3389/fnbeh.2023.1147693

**Published:** 2023-04-04

**Authors:** Mingliang Tan, Zhou Feng, Hui Chen, Lingxia Min, Huizhong Wen, Hongliang Liu, Jingming Hou

**Affiliations:** ^1^Department of Rehabilitation, Southwest Hospital, Army Medical University, Chongqing, China; ^2^Department of Neurobiology, College of Basic Medical Science, Army Medical University, Chongqing, China

**Keywords:** spinal cord injury (SCI), neuropathic pain, transcranial direct current stimulation (tDCS), microglia, cytokines

## Abstract

**Objective:**

Neuropathic pain is a common complication after spinal cord injury (SCI). Transcranial direct current stimulation (tDCS) has been confirmed to be effective in relieving neuropathic pain in patients with SCI. The aim of this study is to investigate the effect of tDCS on neuropathic pain induced by SCI and its underlying mechanism.

**Materials and methods:**

The SCI model was induced by a clip-compression injury and tDCS stimulation was performed for two courses (5 days/each). The motor function was evaluated by Basso-Beattie-Bresnahan (BBB) score, and the thermal withdrawal threshold was evaluated by the thermal radiation method. The effects of tDCS on the cerebral cortex, thalamus, midbrain, and medulla were detected by the enzyme-linked immunosorbent assay (ELISA) and immunofluorescence.

**Results:**

The results showed that SCI reduced the thermal withdrawal threshold and increased the concentration of inflammatory cytokines in the cortex, thalamus, midbrain, and medulla, including the tumor necrosis factor-α (TNF-α), interleukin-1β (IL-1β), and interleukin-6 (IL-6). In addition, the activation of microglia and the proportion of M1 phenotypic polarization increased significantly in the ventral posterolateral (VPL), ventral tegmental (VTA), and periaqueductal gray (PAG) regions after SCI. After tDCS treatment, the thermal withdrawal threshold and motor function of SCI rats were significantly improved compared to the vehicle group. Meanwhile, tDCS effectively reduced the concentration of pro-inflammatory cytokines in the cortex, thalamus, midbrain, and medulla and increased the concentration of anti-inflammatory cytokines interleukin-10 (IL-10) in the thalamus. In addition, tDCS reduced the proportion of the M1 phenotype of microglia in VPL, VTA, and PAG regions and increase the proportion of the M2 phenotype.

**Conclusion:**

The results suggest that tDCS can effectively relieve SCI-induced neuropathic pain. Its mechanism may be related to regulating the inflammatory and anti-inflammatory cytokines in corresponding brain regions *via* promoting the phenotypic transformation of microglia.

## 1. Introduction

As a common complication of spinal cord injury (SCI), neuropathic pain develops in more than 40–60% of SCI patients, which seriously affects the rehabilitation outcome and quality of daily life (Finnerup et al., 2014). The recommended first-line treatment drugs for neuropathic pain after SCI include pregabalin, gabapentin, and amitriptyline (Guy et al., 2016). However, it was reported that more than 40–60% of SCI patients hadn’t been effectively relieved through drug treatment (Finnerup et al., 2015). Therefore, it is urgent to explore some new and convenient methods for the treatment of neuropathic pain induced by SCI.

Transcranial direct current stimulation (tDCS) is a non-invasive brain stimulation method and has been suggested as a promising therapy for patients with refractory pain in recent years (Yoon et al., 2014). It has been reported that tDCS successfully relieved neuropathic pain in 76% of patients, within which the primary etiological sources of classical neuropathic pain are SCI 65.7% (Zhang et al., 2021). However, the underlying mechanism of tDCS relieving neuropathic pain remains to be further explored.

Microglia are both glia cells and a unique type of mononuclear phagocyte that accounts for approximately 10% of cells in the central nervous system (CNS) (Nguyen et al., 2017; Zheng et al., 2022). Under healthy conditions, microglia help maintain CNS homeostasis (Shen et al., 2019). However, microglia play a double-edged role in the CNS through phenotypic transformation, including pro-inflammatory phenotype (harmful; M1-like phenotype) and anti-inflammatory phenotype (protective; M2-like phenotype). M1-like phenotype microglia secretes pro-inflammatory cytokines and aggravates inflammatory response and neural injury. On the contrary, M2-like phenotype microglia secretes anti-inflammatory cytokines, inhibits inflammatory response, and promotes neuroprotection. Regulating the phenotype of microglia in different brain regions will help to regulate the types of cytokines in corresponding brain rergions (Kwon and Koh, 2020).

In recent years, studies have shown that neuropathic pain is related to microglial synaptic remodeling, especially in the emotional and memory-related brain regions (Inoue and Tsuda, 2018). It also has been reported that tDCS could promote functional recovery by regulating the phenotypic transition of microglia in a rat model of stroke (Braun et al., 2016). Based on the above findings, we speculate that the mechanism of tDCS alleviating neuropathic pain after SCI may be related to regulating the phenotypic transition of microglia to reduce inflammation in some pain-related brain regions.

## 2. Materials and methods

### 2.1. Animal preparation and experimental groups

Thirty female Sprague–Dawley (SD) rats (weighing 230–250 g) were purchased from the animal center of Army Medical University (Chongqing, China). Prior to the experiment, the animals were isolated in a clean cage with adequate food and water, a 12-h light/dark cycle and a constant temperature of 24°C. The thirty rats were randomly divided into sham group (*n* = 10), vehicle group (*n* = 10), and tDCS group (*n* = 10). Animal use protocols were approved by the Animal Care and Use Committee of the Army Medical University (AMUWEC20223969).

### 2.2. SCI model

The SCI model was induced by a clip-compression injury method as described previously (Feng et al., 2021). In short, every groups were anesthetized by intraperitoneal injection of pentobarbital sodium (40 mg/kg). Under aseptic conditions, expose the spine of rats along the central axis of the spine incision, remove T9 vertebral lamina, expose the spinal cord, and the vehicle group and the tDCS group were used a clip (50 g closing force) to completely clamp the spinal cord for 60 s to induce SCI model. The rats in the sham group received the above surgical exposure but did not clamp the spinal cord. All rats received manual bladder care twice a day until bladder control was restored.

### 2.3. Surgery and tDCS

**FIGURE 1:**
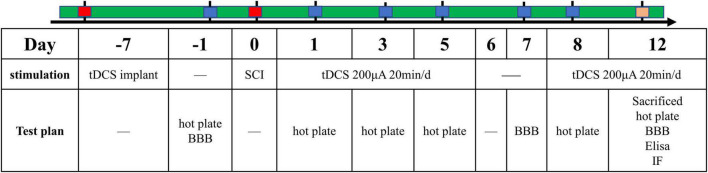
The time schedule of protocols in the present study.

One week before the establishment of the SCI model, we performed electrode implantation in rats of the above three groups. Surgery was performed under anesthetized by intraperitoneal injection of pentobarbital sodium (40 mg/kg). We implanted the cerebral motor cortex with electrodes as described in our previous article (Wen et al., 2017). Briefly, the saline-soaked sponge was wrapped around the end of the copper wire and placed into a plastic tube (inner diameter: 2 mm; length: 1 cm). The copper wire and sponge are secured within the tube using polyacrylate adhesive. Use a stereotaxic instrument to fix the above plastic tube on the skull above the motor cortex (bregma: AP + 0.00 mm, ML 2.00 mm) as the stimulating electrode. The cathode electrode is a large conventional electrode (10 cm^2^) placed in an elastic vest that wraps around the ventral thorax. After the operation, rats were transferred back to their cages and given plenty of food and water. The temporal evolution of the study protocols were summarized (Figure 1).

The above three groups of rats were given stimulation according to the protocols. The sham group and vehicle group were given sham-tDCS stimulation, and the tDCS group was given real tDCS stimulation. The stimulation regimen was repeated daily with 200 μA tDCS for 20 min for 5 days, followed by a 2-day interval, and then the animals received tDCS for 5 more days, resulting in a total of 10 days of tDCS. In order to prevent the sudden change of electric current from damaging the brain, it was used to up or down the current 10 s after the start and before the end of tDCS stimulation. For sham-tDCS, the stimulation electrode was placed in the same position as the real stimulus, but the stimulation duration was only 10 s (Wen et al., 2017).

### 2.4. BBB score

The motor function of the hindlimb was evaluated by Basso-Beattie-Bresnahan (BBB) score (Basso et al., 1995). Rats were placed in a circular platform with a diameter of 2 m and were scored 1 day before the establishment of the SCI model and 1, 3, 5, 7, and 12 days after injury. The BBB score was divided into three stages, with a total score of 21. The assessment was conducted by two independent inspectors who turned a blind eye to the treatment plan.

### 2.5. Thermal hyperalgesia

The thermal hyperalgesia test was carried out according to the previous research method to estimate the heat withdrawal latency (Wen et al., 2017). All rats were placed in a test cage with a glass plate after a 30-min adaptation period, and then the irradiation lamp under the glass plate (52 ± 0.2°C) was irradiated on the plantar surface of the right hind paw. The response time of thermal hyperalgesia was recorded by lifting the hind paw, flicking, or starting to jump as the response point. To avoid tissue damage, the cut-off time was set at 70 s. Each hind paw was irradiated three times with an interval of 5 min each time.

### 2.6. Tissue preparation

Immediately after the stimulation, the rats were deeply anesthetized with pentobarbital sodium (40 mg/kg), and 0.9% sterile saline was perfused into the heart. Immediately after perfusion, the relevant brain regions of appropriate size were dissected on the ice and stored at −80°C for tissue cytokine detection. Another part of rats was perfused with precooled 4% paraformaldehyde after the above saline perfusion. After perfusion, the brain was dissected and fixed in paraformaldehyde overnight. Then the brain was dehydrated in different concentrations of sucrose solution (10, 20, and 30%) and embedded in OCT, and the tissue was cut into frozen sections with a thickness of 18 microns using a slicer (Leica, Wetzlar, Hesse, Germany).

### 2.7. Enzyme-linked immunoabsorbent assay (ELISA)

The brain tissue samples in the cerebral cortex, thalamus, midbrain, and medulla were collected on the last day of tDCS stimulation. ELISA method was used to observe the expression levels of proinflammatory cytokines, including tumor necrosis factor-α (TNF-α), interleukin-1β (IL-1β), interleukin-6 (IL-6), and anti-inflammatory cytokines interleukin-10 (IL-10) in above brain regions. The tissues were homogenized with radioimmunoprecipitation assay (RIPA) lysis buffer, including 1 mM EDTA, 150 mM NaCl, 10 mM Tris (pH 8.0), 1 mM PMSF, and protease inhibitors, and incubated at 4°C for 1 h with shaking. The supernatant was collected by centrifugation at 4°C for 12,000 rpm for 10 min, and then stored at −80°C (Chen et al., 2022). The concentration of cytokines was measured according to the ELISA kit manufacturer’s instructions (Boster, Wuhan, China). The total protein was determined by the BCA method, according to the manufacturer’s kit operation manual (Beyotime, Shanghai, China).

### 2.8. Immunofluorescence and cell counting

After washed with PBS (5 min each). Prepared brain sections were permeated with 0.5% Triton X-100 for 30 min, and blocked with 10% goat serum for 2 h at room temperature, followed by overnight incubation with primary antibody at 4°C. The next day, samples were incubated with the corresponding secondary antibody at room temperature for 2 h. And 4′, 6-diamino-2-phenylindole (DAPI; Boster, Wuhan, China) were counterstained for 5 min. Finally, stained sections were examined, and images were captured using a confocal microscope (LSM-880; Zeiss). The primary antibodies used in the experiment are as follows: goat anti-ionized calcium-binding adaptor molecule (Iba1; 1:200; Abcam, UK), rabbit anti-Iba1 (1:200; GeneTex, Irvine, CA, USA), mouse anti-CD86 (1:200; Santa Cruz, CA, USA), rabbit anti-CD206 (1:200; Abcam, UK). Images from four sections of the brain around the PAG, VTA and VPL were captured using a 20× objective on a Zeiss confocal microscope (Zeiss, LSM780, Germany). Cell numbers were calculated per random microscopic field (100 × magnification). All counts were performed in a blinded fashion.

### 2.9. Quantitative reverse transcription-polymerase chain reaction (qRT-PCR)

Total RNA was extracted from different brain tissues with TRIzol (Takara, Japan) according to the manufacturer’s protocol. The purity and concentration of total RNA in each sample were then evaluated by UV-visible spectrophotometer (NanoDrop2000, Thermo Fisher Scientific, Waltham, MA, USA). Complementary DNA (cDNA) was prepared by using reverse transcription kit (Takara, Japan) and then SYBR Green Premix Pro (Takara, Japan). The CFX-96 real-time PCR detection system (Bio-Rad, Hercules, CA, USA) performs the following reactions: 95°C for 5 min, 40 cycles of 95°C for 5 s, and 60°C for 30 s, following the melting curve. The mRNA levels of target genes were normalized to β-actin expression. Relative quantification of gene expression was performed by the 2^–ΔΔ*CT*^ method. The primer sequences are listed in Table 1.

**TABLE 1:** Real-time PCR primers used in this study.

Target/control gene	Primer sequences
Iba1	F 5′-ATGTCCTTGAAGCGAATGCT-3′ R 5′-TTCTCAAGATGGCAGATCTCTT-3′
CD86	F 5′-GACACCCACGGGATCAATTA-3′ R 5′-GCCTCCTCTATTTCAGGTTCAC-3′
CD206	F 5′-ACTGCGTGGTGATGAAAGG-3′ R 5′-TAACCCAGTGGTTGCTCACA-3′
*β*-actin	F 5′-GTCGTACCACTGGCATTGTG-3′ R 5′-CTCTCAGCTGTGGTGGTGAA-3′

### 2.10. Statistical analysis

SPSS 22.0 software was used to perform statistical analysis. Different behaviors were analyzed by repeated measures analysis of variance (ANOVA), followed by Bonferroni’s *post-hoc* test. One-way ANOVA followed by the Bonferroni’s *post-hoc* test was used for the other multiple comparisons in this study. Values are presented as means ± standard deviation (SD). For all of the tests, three levels of significance were determined: ***p < 0.001, **p < 0.01, and *p < 0.05.

## 3. Results

### 3.1. tDCS relieves neuropathic pain induced by spinal cord injury

**FIGURE 2:**
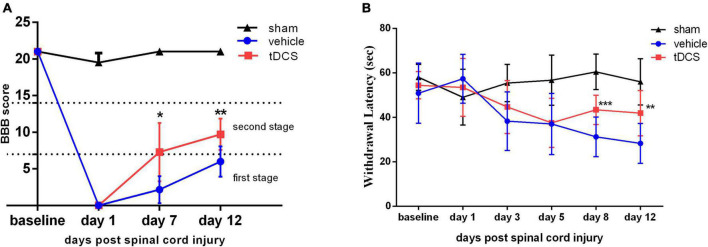
tDCS promotes the recovery of behavioral tests after SCI. Statistical analysis of **(A)** the Basso-Beattie-Bresnahan (BBB) score and **(B)** the Withdrawal latency for the sham, vehicle, and tDCS groups. tDCS group vs. Vehicle group, ***p < 0.001, **p < 0.01, *p < 0.05, *n* ≥ 7.

We used BBB score to evaluate the effect of tDCS on the recovery of motor function in SCI rats. As shown in Figure 2A, on the 7th days (*F* = 54.58, df = 2, 14, *p* = 0.017) and 12th days (*F* = 83.58, df = 2, 14, *p* = 0.005) after SCI, BBB scores were significantly higher in the tDCS group compared with the vehicle group and the sham group. BBB score in the vehicle group was in the first stage, while the BBB score in the tDCS group was in the second stage. We also detected the thermal withdrawal threshold before modeling and on the 1st, 3rd, 5th, 8th, and 12th days after SCI (Figure 2B). Before SCI modeling and 1st, 3rd, and 5th days after SCI, there was no significant difference between the tDCS group and vehicle group. On the 8th days (*F* = 48.96, df = 2, 40, *p* < 0.001) and 12th (*F* = 27.55, df = 2, 40, *p* = 0.001) days after stimulation, tDCS could significantly improve the thermal withdrawal threshold of SCI model rats when compared with the vehicle group.

### 3.2. tDCS regulates the content of inflammatory cytokines in related brain regions

**FIGURE 3:**
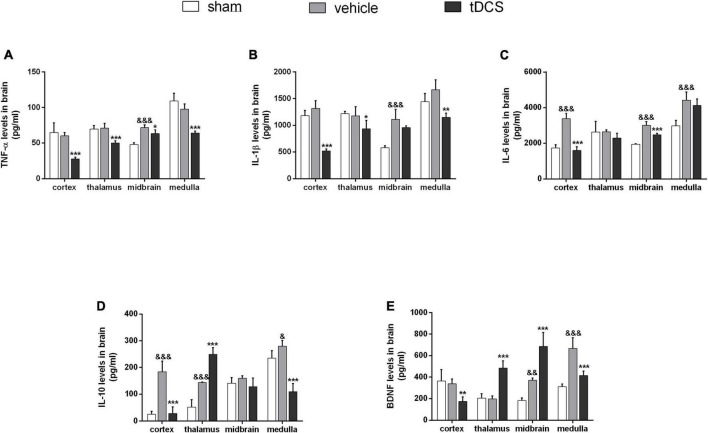
Effect of tDCS on the contents of inflammatory cytokines in cortex, thalamus, midbrain, and medulla of rats with SCI. Statistical analysis results of **(A)** TNF-a, **(B)** IL-ß, **(C)** IL-6, **(D)** IL-10, and **(E)** BDNF for three groups in cortex, thalamus, midbrain, and medulla of rats with SCI. vehicle group vs. sham group, ^&&&^*p* < 0.001, ^&&^*p* < 0.01, and ^&^*p* < 0.05; tDCS group vs. vehicle group, ***p < 0.001, **p < 0.01, and *p < 0.05, *n* = 4.

After completing all courses of stimulation, the rats were taken immediately, and the contents of inflammatory cytokines TNF-a, IL-1ß, IL-6, and anti-inflammatory cytokines IL-10 in different brain regions were detected by ELISA. As shown in Figure 3A, the level of TNF-a in the midbrain was significantly increased in SCI rats (*F* = 34.94, df = 2, 9, *p* < 0.001), and tDCS could significantly decrease the content of TNF-a in the cortex (*F* = 22.25, df = 2, 9, *p* < 0.001), thalamus (*F* = 20.66, df = 2, 9, *p* < 0.001), midbrain (*F* = 34.94, df = 2, 9, *p* = 0.017), and medulla (*F* = 35.10, df = 2, 9, *p* < 0.001). As shown in Figure 3B, the level of IL-1ß in the midbrain was increased in SCI rats (*F* = 21.73, df = 2, 9, *p* < 0.001), and tDCS could decrease the levels of IL-1ß in the cortex (*F* = 68.38, df = 2, 9, *p* < 0.001), thalamus (*F* = 5.00, df = 2, 9, *p* = 0.032), and medulla (*F* = 11.98, df = 2, 9, *p* = 0.001). As shown in Figure 3C, the levels of IL-6 in the cortex (*F* = 61.73, df = 2, 9, *p* < 0.001), midbrain (*F* = 53.89, df = 2, 9, *p* < 0.001), and medulla (*F* = 15.79, df = 2, 9, *p* < 0.001) were increased in SCI rats, and tDCS could decrease the levels of IL-6 in the cortex (*F* = 61.73, df = 2, 9, *p* < 0.001) and midbrain (*F* = 53.89, df = 2, 9, *p* < 0.001). As shown in Figure 3D, as an anti-inflammatory cytokine, the levels of IL-10 in the cortex (*F* = 44.07, df = 2, 9, *p* < 0.001), thalamus (*F* = 81.17, df = 2, 9, *p* < 0.001), and medulla (*F* = 43.60, df = 2, 9, *p* = 0.042) were increased in SCI rats, and tDCS could further increase the level of IL-10 in the thalamus (*F* = 81.17, df = 2, 9, *p* < 0.001). Brain-derived neurotrophic factor (BDNF) plays an important role in protecting the central nervous system, As shown in Figure 3E, the levels of BDNF in the midbrain (*F* = 43.83, df = 2, 9, *p* = 0.007) and medulla (*F* = 33.18, df = 2, 9, *p* < 0.001) were increased in SCI rats, and tDCS could increase the levels of BDNF in the thalamus (*F* = 46.56, df = 2, 9, *p* < 0.001) and midbrain (*F* = 43.83, df = 2, 9, *p* < 0.001).

### 3.3. tDCS induces phenotypic transformation of microglia in the VPL

**FIGURE 4:**
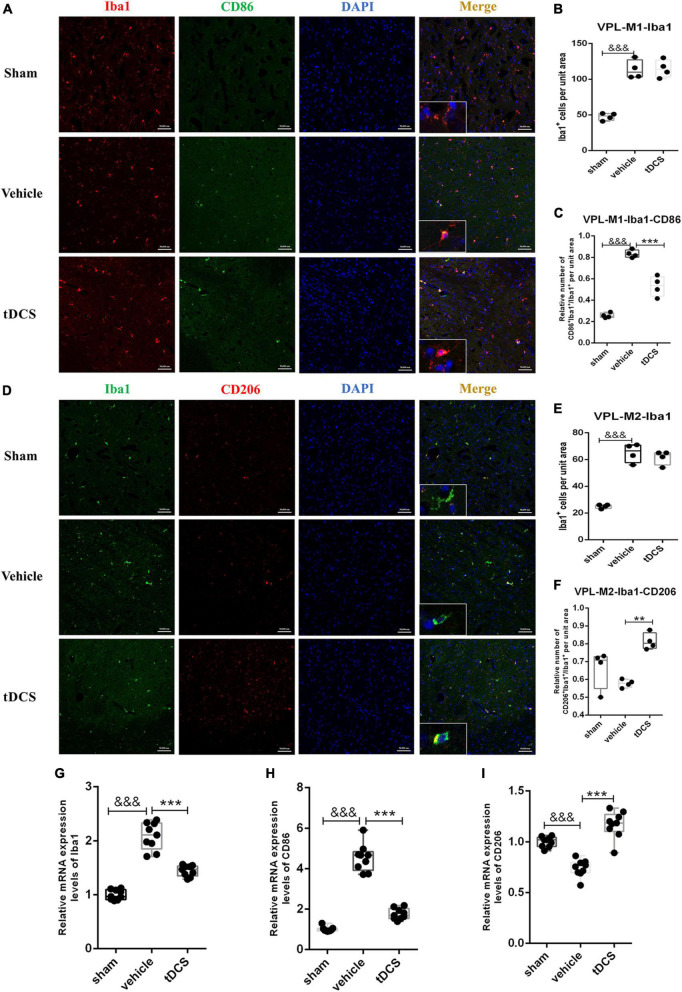
tDCS alters the direction of microglial polarization in the thalamic VPL region. **(A)** Iba1 and CD86 protein immunofluorescence co-staining in three groups. Iba1 protein was stained with red fluorescence, and CD86 protein was stained with green fluorescence. Nuclei were labeled with blue fluorescence. Merged pictures show positive cells. Bar = 50 μm. **(B)** Statistical analysis results of the expression of Iba1 in microglia of each group. **(C)** Statistical analysis results of the proportion of CD86 and Iba1 positive cells to Iba1 positive cells in microglia of each group. **(D)** Iba1 and CD206 protein immunofluorescence co-staining in three groups. Iba1 protein was stained with green fluorescence, and CD206 protein was stained with red fluorescence. nuclei were labeled with blue fluorescence. Merged pictures show positive cells. Bar = 50 μm. **(E)** Statistical analysis results of the expression of Iba1 in microglia of each group. **(F)** Statistical analysis results of the proportion of CD206 and Iba1 positive cells to Iba1 positive cells in microglia of each group. **(G–I)** Relative mRNA expression levels of Iba1, CD86, and CD206 in each groups, as detected by qRT-PCR. Vehicle group vs. sham group, ^&&&^*p* < 0.001; tDCS group vs. vehicle group, ***p < 0.001 and **p < 0.01, *n* = 4.

To detect the activation of microglia in the ventral posterolateral (VPL) region of the thalamus, we detected microglial activation marker Iba1, M1 phenotypic marker CD86, and M2 phenotypic marker CD206 by immunofluorescence staining after stimulation. It was found that SCI caused the morphology of microglia to become amoeba-like, but tDCS did not reverse the above morphological changes (insets of Figures 4A, D). Meanwhile, SCI could significantly increase the expression of Iba1 in the VPL region [Figure 4B (*F* = 36.64, df = 2, 8, *p* < 0.001), and Figure 4E (*F* = 75.82, df = 2, 9, *p* < 0.001)]. Although tDCS treatment did not decrease the expression of Iba1, it decreased the expression of M1 marker CD86 [Figure 4C (*F* = 72.09, df = 2, 8, *p* < 0.001)] and increased the expression of M2 marker CD206 [Figure 4F (*F* = 11.76, df = 2, 9, *p* = 0.001)]. At the same time, we carried out qRT-PCR detection of the tissue in VPL and found that SCI could increase the mRNA levels of Iba1 (*F* = 96.73, df = 2, 24, *p* < 0.001) and CD86 (*F* = 170.40, df = 2, 24, *p* < 0.001), and decrease the mRNA level of CD206 (*F* = 47.43, df = 2, 24, *p* < 0.001), but the mRNA levels of Iba1 (*F* = 96.73, df = 2, 24, *p* < 0.001), and CD86 (*F* = 170.40, df = 2, 24, *p* < 0.001) were decreased, and increase the mRNA levels of CD206 (*F* = 47.43, df = 2, 24, *p* < 0.001) after tDCS (Figures 4G–I). The above results showed that tDCS could regulate the direction of microglial polarization (from M1 to M2) without changing the activation of microglia in the VPL region.

### 3.4. tDCS induces phenotypic transformation of microglia in the PAG

**FIGURE 5:**
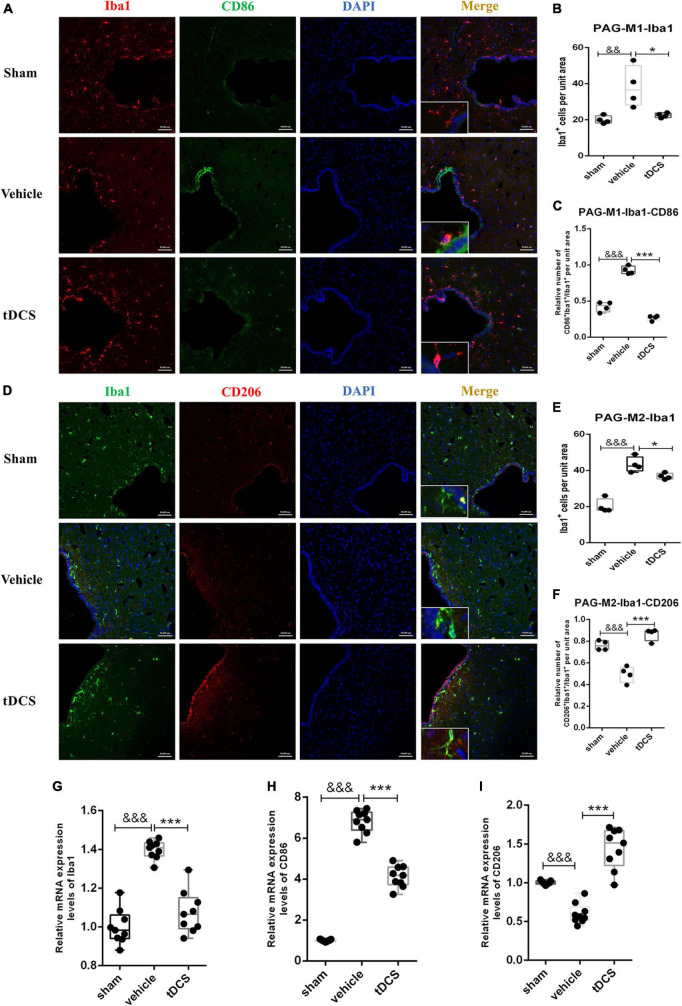
tDCS alters microglial polarization direction in the midbrain PAG region. **(A)** Iba1 and CD86 protein immunofluorescence co-staining in three groups. Iba1 protein was stained with red fluorescence, and CD86 protein was stained with green fluorescence. nuclei were labeled with blue fluorescence. Merged pictures show positive cells. Bar = 50 μm. **(B)** Statistical analysis results of the expression of Iba1 in microglia of each group. **(C)** Statistical analysis results of the proportion of CD86 and Iba1 positive cells to Iba1 positive cells in microglia of each group. **(D)** Iba1 and CD206 protein immunofluorescence co-staining in three groups. Iba1 protein was stained with green fluorescence, and CD206 protein was stained with red fluorescence. Nuclei were labeled with blue fluorescence. Merged pictures show positive cells. Bar = 50 μm. **(E)** Statistical analysis results of the expression of Iba1 in microglia of each group. **(F)** Statistical analysis results of the proportion of CD206 and Iba1 positive cells to Iba1 positive cells in microglia of each group. **(G–I)** Relative mRNA expression levels of Iba1, CD86, and CD206 in each groups, as detected by qRT-PCR. Vehicle group vs. sham group, ^&&&^*p* < 0.001 and ^&&^*p* < 0.01; tDCS group vs. vehicle group, ***p < 0.001 and *p < 0.05, *n* = 4.

Similarly, we performed immunofluorescence co-staining on the periaqueductal gray (PAG) region of the midbrain. The main morphology of microglia in PAG was amoebic after SCI, and tDCS increased microglia branching (insets of Figures 5A, D). SCI could increase the activation of microglia in the PAG region, which is mainly characterized by increasing the proportion of M1 phenotypic microglia [Figure 5B (*F* = 7.40, df = 2, 8, *p* = 0.007), and Figure 5C (*F* = 129.14, df = 2, 8, *p* < 0.001)] and decreasing the proportion of M2 phenotypic microglia [Figure 5E (*F* = 47.63, df = 2, 9, *p* < 0.001) and Figure 5F (*F* = 40.17, df = 2, 9, *p* < 0.001)]. After tDCS treatment, the activation of microglia in the PAG region decreased [Figure 5B (*F* = 7.40, df = 2, 8, *p* = 0.021) and Figure 5E (*F* = 47.63, df = 2, 9, *p* = 0.025)], showing a decrease in the proportion of M1 phenotypic microglia [Figure 5C (*F* = 129.14, df = 2, 8, *p* < 0.001)] and an increase in the proportion of M2 phenotypic microglia [Figure 5F (*F* = 40.17, df = 2, 9, *p* < 0.001)]. The tissues in PAG were detected by qRT-PCR and found that SCI would increase the mRNA levels of Iba1 (*F* = 54.51, df = 2, 24, *p* < 0.001) and CD86 (*F* = 401.08, df = 2, 24, *p* < 0.001), and decrease the mRNA level of CD206 (*F* = 56.31, df = 2, 24, *p* < 0.001), but the mRNA levels of Iba1 (*F* = 54.51, df = 2, 24, *p* < 0.001), CD86 (*F* = 401.08, df = 2, 24, *p* < 0.001) were decreased, and increase the mRNA levels of CD206 (*F* = 56.31, df = 2, 24, *p* < 0.001) after tDCS (Figures 5G–I). The above results suggest that SCI could induce the activation of microglia in the PAG region, and the M1 phenotype is the main polarization direction. Meanwhile, tDCS could reduce the activation of microglia in the PAG region, inhibit their polarization to the M1 phenotype, and promote the polarization to the M2 phenotype.

### 3.5. tDCS induces phenotypic transformation of microglia in the VTA

**FIGURE 6:**
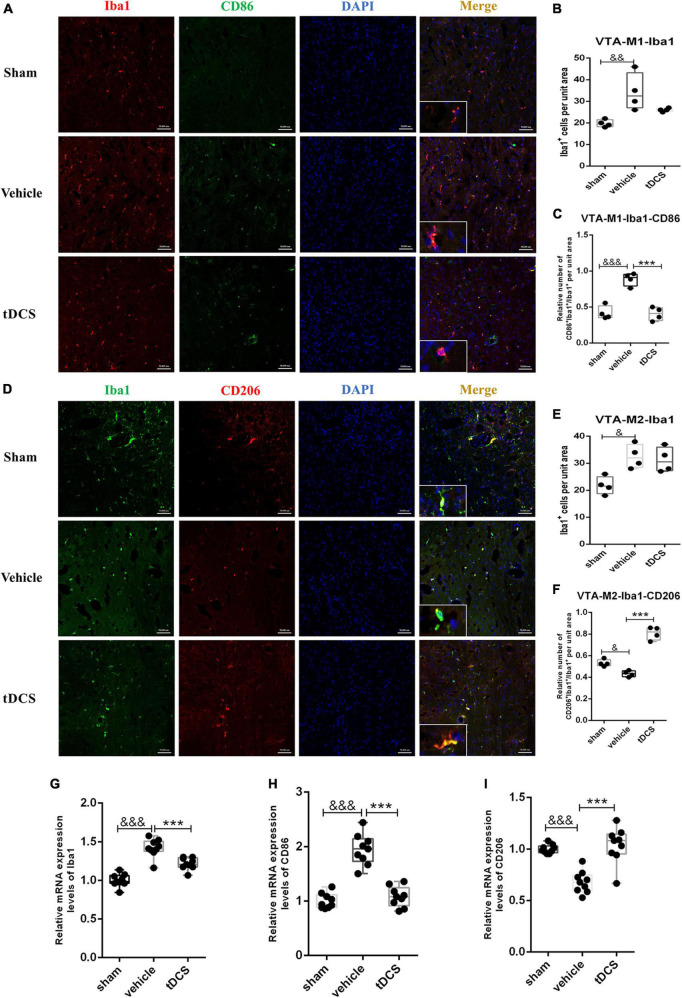
tDCS alters microglial polarization direction in the midbrain VTA region. **(A)** Iba1 and CD86 protein immunofluorescence co-staining in three groups. Iba1 protein was stained with red fluorescence, and CD86 protein was stained with green fluorescence. Nuclei were labeled with blue fluorescence. Merged pictures show positive cells. Bar = 50 μm. **(B)** Statistical analysis results of the expression of Iba1 in microglia of each group. **(C)** Statistical analysis results of the proportion of CD86 and Iba1 positive cells to Iba1 positive cells in microglia of each group. **(D)** Iba1 and CD206 protein immunofluorescence co-staining in three groups. Iba1 protein was stained with green fluorescence, and CD206 protein was stained with red fluorescence. Nuclei were labeled with blue fluorescence. Merged pictures show positive cells. Bar = 50 μm. **(E)** Statistical analysis results of the expression of Iba1 in microglia of each group. **(F)** Statistical analysis results of the proportion of CD206 and Iba1 positive cells to Iba1 positive cells in microglia of each group. **(G–I)** Relative mRNA expression levels of Iba1, CD86 and CD206 in each groups, as detected by qRT-PCR. Vehicle group vs. sham group, ^&&&^*p* < 0.001, ^&&^*p* < 0.01 and ^&^*p* < 0.05; tDCS group vs. vehicle group, ***p < 0.001, *n* = 4.

The ventral tegmental area (VTA) is an important area of dopamine neurons emitted from the midbrain region and a crucial part of pain relief. In this study, we included it in the detection of the midbrain region. Microglia in the VTA region mainly showed amoeba morphology after SCI (insets of Figures 6A, D). SCI could increase the activation of microglia in the VTA region [Figure 6B (*F* = 6.13, df = 2, 8, *p* = 0.009) and Figure 6E (*F* = 4.72, df = 2, 7, *p* = 0.03)], which is mainly characterized by increasing the proportion of M1 phenotypic microglia [Figure 6C (*F* = 29.96, df = 2, 8, *p* < 0.001)]. After tDCS treatment, the proportion of M1 phenotypic microglia decreased [Figure 6C (*F* = 29.96, df = 2, 8, *p* < 0.001)], and the proportion of M2 phenotypic microglia increased [Figure 6F (*F* = 58.89, df = 2, 7, *p* < 0.001)]. At the same time, we adopted qRT-PCR detection of the tissue in VTA and found that SCI could increase the mRNA levels of Iba1 (*F* = 43.97, df = 2, 24, *p* < 0.001) and CD86 (*F* = 55.51, df = 2, 24, *p* < 0.001), and decrease the mRNA level of CD206 (*F* = 24.54, df = 2,24, *p* < 0.001), but the mRNA levels of Iba1 (*F* = 43.98, df = 2, 24, *p* < 0.001), and CD86 (*F* = 55.51, df = 2, 24, *p* < 0.001) were decreased, and increased the mRNA levels of CD206 (*F* = 24.54, df = 2, 24, *p* < 0.001) after tDCS (Figures 6G–I). The above results suggested that the effect of tDCS on VTA is mainly through changing the polarization direction of microglia.

## 4. Discussion

**FIGURE 7:**
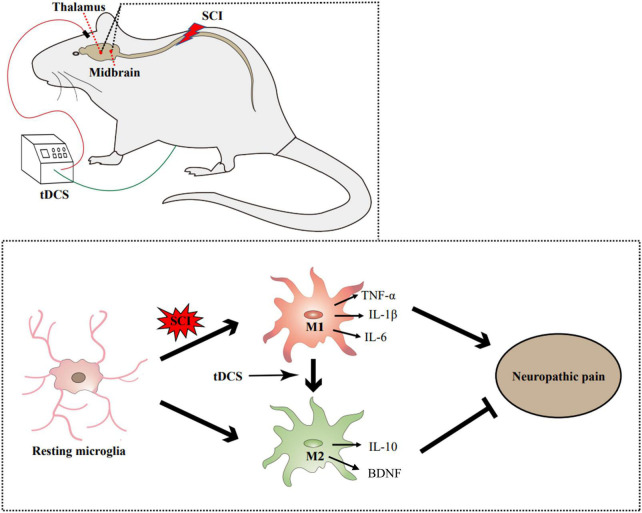
Illustrative mechanism for tDCS to alleviate the neuropathic pain induced by SCI. SCI can promote the activation of microglia and transform into the M1 phenotype. tDCS can change the polarization direction of microglia in the thalamus and midbrain. By reducing the proportion of M1 phenotype and increasing the proportion of m2 phenotype, tDCS can reduce the content of proinflammatory cytokines (TNF-α, IL-1β, and IL-6), increase the content of anti-inflammatory cytokine (IL-10) and neurotrophic factors (BDNF), and finally alleviate the neuropathic pain induced by SCI.

In this study, we found that stimulation of the cerebral motor cortex by tDCS can act on the midbrain and thalamus, thereby relieving neuropathic pain in a rat model of SCI. The mechanism is related to inhibiting the pro-inflammatory activation of microglia to M1 phenotype and promoting the polarization of microglia to M2 anti-inflammatory phenotype, thus reducing the content of pro-inflammatory cytokines in the midbrain and thalamus. In general, the current study provides a molecular biological mechanism for the clinical use of tDCS, which demonstrated that tDCS could alleviate SCI-induced neuropathic pain by regulating the microglial phenotype in pain-related brain regions, and then regulating the content of inflammatory cytokines (Figure 7).

According to clinical reports, more than 2/3 of patients with SCI suffer from chronic neuropathic pain, but there is still a lack of particularly effective intervention (Shiao and Lee-Kubli, 2018). tDCS is a non-invasive brain stimulation technique that was used for the modulation of CNS excitability (Woods et al., 2016). Numerous studies have reported that it could be used to treat neurological disorders (Begemann et al., 2020; Fregni et al., 2021) and chronic pain (De Souza et al., 2021; Knotkova et al., 2021). Since tDCS was first used in the clinical treatment of neuropathic pain after SCI in 2006, its effectiveness has been reported in many studies in recent years (Li et al., 2021). However, the mechanism of tDCS relieving neuropathic pain is not completely clear.

The mechanism of neuropathic pain is complex, and various changes in molecules and plasticity of the CNS may contribute to the development and maintenance of neuropathic pain, in which the up-regulation or down-regulation of chemokines is an important predisposing factor (Widerstrom-Noga, 2017). In this study, we examined cytokines in multiple brain regions after SCI, including the cortex, thalamus, midbrain, and medulla. We found that SCI could increase pro-inflammatory cytokines mainly in the cortex, midbrain, and medulla. As an important decision-making center, the cortex can receive and process information from multiple brain areas and regulate various physiological functions, playing an important role in both pain and comorbidity associated with chronic pain (Jang et al., 2021). The thalamus is the main channel for transmitting various sensory information from the periphery to the cortex (Liang et al., 2020). In addition, the midbrain and medulla also play important roles in neuropathic pain (Yen and Lu, 2013; Huang et al., 2019; Mokhtari et al., 2020). We found that the content of inflammatory factors was inhibited to some extent in the above brain regions after tDCS treatment, including the TNF-α, IL-1β, and IL-6. TNF-a has been confirmed to regulate synaptic plasticity and induce symptoms such as thermal hyperalgesia. Similarly, IL-1β and IL-6 are related to the pathogenesis of neuropathic pain (Liu et al., 2019). Controlling the content of the above inflammatory factors is beneficial in relieving the occurrence of neuropathic pain. As an effective anti-inflammatory and neuroprotective cytokine, IL-10 plays an active role in relieving neuropathic pain (Durante et al., 2021). In our study, we found that tDCS can increase the content of IL-10 in the thalamus. Taken together, our study demonstrated that tDCS exerts its analgesic effect might by reducing the content of pro-inflammatory factors in the midbrain region and increasing the content of anti-inflammatory factors in the thalamus.

As the innate immune cells of the CNS, microglia play an important role in the balance of the microenvironment of the central system (Inoue and Tsuda, 2018). Under different stimulation conditions, it will polarize into a pro-inflammatory M1 phenotype with surface antigen CD86 or an anti-inflammatory M2 phenotype with surface antigen CD206, and secrete pro-inflammatory factors TNF-α, IL-1β, IL-6, and IL-12 or anti-inflammatory factors IL-4, IL-10, and TGF-β (Orihuela et al., 2016) and Neurotrophic factors BDNF (Zhou et al., 2020) that its content is positively correlated with heat pain threshold (Sorkpor et al., 2021). An increasing number of animal experiments have found that regulating the phenotype of different microglia can alter the expression of cytokines, resulting in different outcomes after nerve injury (Zeng et al., 2019). Increasing the proportion of M2 phenotypic microglia will help to increase the level of anti-inflammatory cytokines and BDNF in the surrounding tissue. Studies have confirmed that anti-inflammatory cytokines play an important role in relieving neuropathic and other chronic pain (Sommer et al., 2018). In this study, we found that SCI could induce the activation of microglia in the VPL, VTA, and PAG regions, and the M1 phenotype is the main polarization direction. The VPL nucleus of the thalamus is one of the most important regions in the CNS and has been shown to play a crucial role in pain signaling (Naderi et al., 2014). Studies have shown that deep brain electrical stimulation VPL can effectively relieve neuropathic pain (Kim et al., 2012). The VTA and PAG were the key elements of the descending pain control system in the midbrain areas (Gao et al., 2020; Armin et al., 2021). After stimulation, we found that tDCS could inhibit microglia polarization to the M1 phenotype and promote polarization to the M2 phenotype in the above brain regions. These results indicated that tDCS stimulation alleviates the neuropathic pain induced by SCI by regulating the phenotype of microglia in VPL, VTA, and PAG regions of the brain.

It is worth noting that recent studies have shown activation of microglia is often accompanied by morphological changes (Lucarini et al., 2020; Micheli et al., 2021). In this study, we also found that SCI could cause amoeba-like microglia in related brain regions. After stimulation, tDCS did not significantly change the morphology of microglia in VPL and VTA regions. This finding is similar to a neuropathic pain study, which also found the a9a10nAChR antagonist a-conotoxin RgIA (RglA) did not change the morphology of microglia while relieving chronic constriction injury pain (Di Cesare Mannelli et al., 2014). This extensive cellular reorganization coincides with an abrupt change in a functional state, which enables microglia to patrol injured neural tissue and rapidly make physical contact with many cells (Pacini et al., 2016). Therefore, tDCS does not change the morphology of microglia in the related brain regions, which will benefit the combination of microglia with damaged neurons and maintain the homeostasis of the nervous system.

## 5. Conclusion

In this study, we demonstrated that tDCS could effectively relieve neuropathic pain induced by SCI in rats. The mechanism of pain relief may be related to tDCS can reduce the content of proinflammatory cytokines, increase the content of anti-inflammatory cytokines, and neurotrophic factors in the thalamus and midbrain by regulating the polarization direction of microglia. In the following research, we will focus on exploring the underlying mechanism of tDCS regulating the phenotypic transformation of microglia, thus finding new targets for clinical treatment of neuropathic pain.

## Data availability statement

The original contributions presented in this study are included in the article/supplementary material, further inquiries can be directed to the corresponding authors.

## Ethics statement

The animal study was reviewed and approved by the Laboratory Animal Welfare and Ethics Committee of the Third Military Medical University.

## Author contributions

MT and JH designed the research, wrote and edited the manuscript. ZF, HW, and HL designed the research. MT, HC, and LM performed the experiments and analyzed the data. All authors contributed to the article and approved the submitted version.
